# Improving the Accuracy of Density Functional Theory (DFT) Calculation for Homolysis Bond Dissociation Energies of Y-NO Bond: Generalized Regression Neural Network Based on Grey Relational Analysis and Principal Component Analysis

**DOI:** 10.3390/ijms12042242

**Published:** 2011-04-01

**Authors:** Hong Zhi Li, Wei Tao, Ting Gao, Hui Li, Ying Hua Lu, Zhong Min Su

**Affiliations:** 1 Institute of Functional Material Chemistry, Faculty of Chemistry, Northeast Normal University, Changchun, 130024, China; E-Mails: lihz857@nenu.edu.cn (H.Z.L.); taow587@nenu.edu.cn (W.T.); 2 School of Computer Science and Information Technology, Northeast Normal University, Changchun, 130017, China; E-Mails: gaot080@nenu.edu.cn (T.G.); lihui@nenu.edu.cn (H.L.)

**Keywords:** Y-NO bond, homolysis bond dissociation energy, density functional theory, grey relational analysis, principal component analysis, generalized regression neural network

## Abstract

We propose a generalized regression neural network (GRNN) approach based on grey relational analysis (GRA) and principal component analysis (PCA) (GP-GRNN) to improve the accuracy of density functional theory (DFT) calculation for homolysis bond dissociation energies (BDE) of Y-NO bond. As a demonstration, this combined quantum chemistry calculation with the GP-GRNN approach has been applied to evaluate the homolysis BDE of 92 Y-NO organic molecules. The results show that the ull-descriptor GRNN without GRA and PCA (F-GRNN) and with GRA (G-GRNN) approaches reduce the root-mean-square (RMS) of the calculated homolysis BDE of 92 organic molecules from 5.31 to 0.49 and 0.39 kcal mol^−1^ for the B3LYP/6-31G (d) calculation. Then the newly developed GP-GRNN approach further reduces the RMS to 0.31 kcal mol^−1^. Thus, the GP-GRNN correction on top of B3LYP/6-31G (d) can improve the accuracy of calculating the homolysis BDE in quantum chemistry and can predict homolysis BDE which cannot be obtained experimentally.

## 1. Introduction

Nitric oxide (NO) is an important signaling and effector molecule that is key to many physiological functions of the body (e.g., blood pressure regulation, immune system and nerve conduction), and plays a vital role in the regulation of [1–9]. NO has a high chemical activity and rarely exists in the form of free radical in the body. It becomes stable by binding itself with the carrier molecules in the body in specific binding sites that enable its storage, transfer and release.

Inspired by the concepts of the “proton affinity” and “electron affinity”, Cheng *et al.* proposed the concept of NO affinity which can be defined as the measure of the strength for a receptor (X) to bind with the NO group [10]. It is characterized using the energies of the Y-NO bond (Y is the atom in the carrier molecules to which the NO group is attached, Y = N, S, O, C) in two different ways:

(1) $$X\;-\;NO\;\to \;X^{•}\;+\;NO^{•}$$

(2) $$X\;-\;NO\;\to \;X^{-}\;+\;NO^{+}$$

The first reaction represents the homolysis which is chemical bond dissociation of a neutral molecule generating two free radicals. The energy during the reaction is referred to as homolysis Bond Dissociation Energy (BDE).The second reaction represents the heterolysis which is chemical bond cleavage of a neutral molecule generation an anions or cations. The energy during the reaction is referred to heterolysis BDE. In recent years, Cheng *et al.* developed a simple experimental approach to measure the homolysis and heterolysis bond dissociation energies (BDE) of the Y-NO (Y = C, N, O, S) bond in solution [10–20]. Experimental data show that the heterolysis energy of Y-NO bond is generally higher than the homolysis BDE of Y-NO bond which implies that it is easier for the NO carrier to release NO than NO^−^ and NO^+^. The carrier molecule is a potential free radical to bond with NO. The study of the heterolysis and homolysis BDE of the carrier molecules containing Y-NO (Y = C, N, O, S) bond helps measure the bonding and release capacity of NO in the body and understand and predict the transfer direction and mechanism of NO in the body.

Quantum chemistry approaches are not only limited to the level of experimental validation, but also can predict the BDE without experimental results or with uncertain experimental. The main reason leading to this limitation is the similarity between the computational approaches. Therefore, in recent 10 years, many statistical approaches have been used to improve the accuracy of quantum chemistry. First, the molecular properties are obtained from the calculation of quantum chemistry approaches and then the statistical approaches are applied to establish the relation between the experimental and calculated values. These statistical improvements include linear approaches, such as multiple linear regression [21], and nonlinear approaches, such as neural networks, *etc.* [22–24]. Although multiple linear regression approach is simple and intuitive, neural networks can better solve complex nonlinear problems which are difficult to model mathematically given the same physical parameters [25–27]. If the training of the neural networks is based on the back propagation (BP) algorithm, it is vulnerable to the slow convergence rate, and gets stuck at the local minimum points [28]. The genetic algorithm is an efficient global search approach which has been adopted in problems with a large search state-space to explore the globally optimal results. Therefore, the genetic algorithm can be used to optimize the weights of neural network [29].

Roman M. Balabin *et al*. estimate the density functional theory (DFT) energy with large basis set using lower-level energy values and molecular descriptors [30]. A total of 208 different molecules were used for the artificial neural network (ANN) training, cross validation, and testing by applying BLYP, B3LYP, and BMK [31] density functionals. An expected error, mean absolute deviation, ANN approximation to DFT energies was 0.6 ±0.2 kcal mol^−1^. Wu and Xu proposed the X1 approach that combines the DFT (B3LYP) with the neural network correction for an accurate prediction of formation heat [25]. An error close to the G3 approach (1.34 *versus* 1.05 kcal mol^−1^ for the G3/99 molecule set) was reached.

Chen and co-workers proposed a DFT-NEURON approach to establish the quantitative relationship between the experimental data and the results computed from the first main principle [23]. This relationship was then used to reduce the error margin of the values of the computed absorption energy [32]. With the TDDFT/B3LYP/6-31G (d) approaches, the root-mean-square (RMS) for the absorption energies in 60 organic molecules was reduced from 0.33 to 0.09 eV. Recently, our research group proposed a successful improvement approach based on genetic algorithm and neural network (GANN) to correct the absorption energies of 150 organic molecules [33]. In addition, we also proposed a least squares support vector machine approach to correct the absorption energies of 160 organic molecules [34].

There are mainly two factors affecting the accuracy of the calculation of the homolysis BDE: (1) the selection of molecular descriptors and pretreatment; (2) the statistical approaches. Some researchers only focus on the statistical approach selection and ignore the molecular descriptors selection and pretreatment. The subjective choice of molecular descriptors, the distribution of the weights, the redundancy in the chosen molecular descriptors and the multiple correlations in molecular descriptors all affect the final results. Therefore, the molecular descriptors selection and pretreatment are significant.

In this paper, the Generalized Regression Neural Network (GRNN) based on the grey relational analysis (GRA) and principal component analysis (PCA) (GP-GRNN) approach is proposed to improve the accuracy of calculating the homolysis BDE of 92 organic molecules. The DFT B3LYP/6-31G (d) approach is first applied to optimize the carrier molecules and calculate their frequency in order to obtain the homolysis BDE value and relevant molecular descriptors of the Y-NO (Y = C, N, O, S) bond. GRA is used to select the appropriate molecular descriptors. PCA is used to optimize the selected molecular descriptors. Finally, GRNN is used to establish nonlinear model. Then the GP-GRNN is applied to reduce the RMS of homolysis BDE for the 92 organic molecules. The results show that GP-GRNN is a more accurate and informative correction technique in chemical physics.

## 2. Description of Approach

### 2.1. Grey Relational Analysis

If the index characteristics of a system are represented as a reference array *x*_0_ (*n*-dimensional), the array is used as reference index (experimental value of homolysis BDE in this paper). If an array that has multiple characteristics is related to the reference index, then the array is represented as *x_i_*(*i* = 1,2,…,*m*) (*n*-dimensional). To measure the relationship between the reference index and the arrays *x_i_*(*i* = 1,2,…,*m*), the concept of relational coefficient is introduced below:

(3) $$r_{i}\;(k)\;=\;\frac{\underset{i}{\text{min}}\underset{k}{\text{min}}\left|x_{0}\;(k)\;-\;x_{i}\;(k)\right|\;+\;\alpha *\underset{i}{\text{max}}\underset{k}{\text{max}}\;\left|x_{0}\;(k)\;-\;x_{i}\;(k)\right|}{\left|x_{0}\;(k)\;-\;x_{i}(k)\right|+\alpha *\;\underset{i}{\text{max}}\underset{k}{\text{max}}\left|x_{0}\;(k)\;-\;x_{i}\;(k)\right|}\;(i=1,2,\ldots ,m;\;k=1,\;2,\;\ldots n)$$

where *α* is the distinguishing coefficient, *r_i_*(*k*) is the relational coefficient between *x_i_* and *x*_0_ at the *k*th characteristic.

The following equation further processes the relational coefficients to obtain the ɛ*_i_* which is the relation degree between *x_i_* and *x*_0_.

(4) $${ɛ}_{i}\;=\;[r_{i}(1)\;+\;r_{i}(2)\;+\;\cdots +\;r_{i}(n)]/n$$

ɛ*_i_* is the average of the relational coefficients [35–37].

### 2.2. Principal Component Analysis

PCA is a multivariate statistical approach used for reducing variables in the data [38]. Its basic content is to convert a set of original indices into a set of comprehensive indices which are new and uncorrelated with one another to replace the original. According to the actual needs, it is likely to select several fewer comprehensive indices which can reflect the information of original indices as much as possible to represent the total indices of original variables to achieve the purpose of variable reduction.

PCA is aimed at compressing the number of variables and making the model reflect the real situation better by using fewer variables to explain most of the variables in the original data and eliminating the redundancy. This means to convert a number of highly correlated variables into new, fewer and independent of one another variables, *i.e.*, principal component which can explain most of the variance of the original data. This approach can erase the collinearity existing in the original variables, and overcome the problems, such as instability of calculation, ill-conditioned matrix and so on and so forth.

The following part shows the approach and calculation steps that PCA adopts to determine the weight.

We suppose that the number of sample (organic molecules) is *n* and the number of indicators’ (molecular descriptors) value in each sample is *m*. Then, we can organize the experimental data into a matrix.

(5) $$X\;=\;[X_{ij}](i=1,2,\ldots ,n;\;j=1,2,\ldots ,m)$$

(a) Standardize the original data

The indicator in each sample is converted into the standardized indicator X^*^_j_ according to the standardized Equation 6. Therein, *X_j_* and *S_j_* is respectively are the mean and standard deviation of *X_j_*. The mean of X^*^_j_ is 0 and the variance is 1.

(6) $$X_{ij}^{*}\;=\;\frac{X_{ij}\;-\;{\bar{X}}_{j}}{S_{j}}(i=1,2,\ldots ,n;\;j=1,2,\ldots ,m)$$

(b) Calculate the correlation coefficient *r_ij_,* of each standardized indicator X^*^_j_ and write down the matrix of correlation coefficient: *R* = [*r_ij_*]_m__*__m_. Therein,

(7) $$r_{ij}\;=\;\frac{1}{n-1}\sum_{t=1}^{n}X_{ti}X_{tj}(i,j\;=\;1,2,\ldots ,m)$$

(c) Calculate the eigenvalue *λ*_i_ (*I* = 1,2,…,*m*) of related matrix *R* and then, arrange the eigenvalue *λ_i_* according to the descending order *λ*_1_ ≥ *λ*_2_ ≥ *λ*_3_ ≥ … ≥ *λ_m_* ≥ 0. $\alpha_{i}\;=\;\frac{\lambda_{i}}{m}$ is called the variance contribution of the principal component *Z_i_*, *i.e*., the weight of the principal component *Z_i_*.

### 2.3. Generalized Regression Neural Network

**Figure 1.**
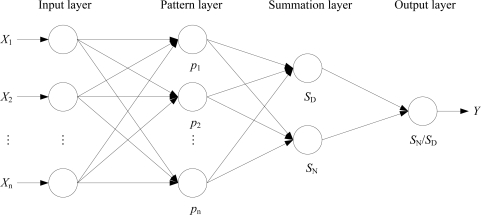
Structure of generalized regression neural network (GRNN).

GRNN was proposed by the American scholar D. F. Specht [39]. The approach uses vertical basis function as the basis of the hidden units to form the hidden layers. The hidden layers (include pattern layer and summation layer) transform the input vectors from the low-dimensional input data into a high dimensional space so that the problem can be separated linearly in the high dimensional space. It is good at function approximation and the network finally converges to the optimized regression plane which contains the most samples. It can predict well, even with very few sample data, and can handle instability in the data. The structure of GRNN is composed of four layers, input layer, pattern layer, summation layer and output layer (Figure 1).

The output is Y which corresponds to the net input *X =* [*x*_1_,*x*_2_,*…*,*x_p_*]^T^. The number of neurons in the input layer is equal to the dimensions of input vector *p* in the study sample. The number of neurons in pattern layer is equal to the number of study sample *n*. The transfer function of neuron n is

(8) $$p_{i}\;=\;\text{exp}\left[-\frac{{\left(X\;-\;X_{i}\right)}^{T}\;(X\;-\;X_{i})}{2\delta^{2}}\right](i\;=\;1,2,\ldots n)$$

therein, *X* is the network’s input variable and *δ* is the smoothing factor which determines the shape of function. The larger the value is, the smoother the function is. *X_i_* is the corresponding study sample of neuron *i.* Each unit in the pattern layer corresponds to a training sample and the Gaussian function is treated as the activation of kernel function. Two types of neurons are used for summation in the summation layer, one is

(9) $$X\;=\;[X_{ij}](i\;=\;1,2,\ldots ,n;\;j\;=\;1,2,\ldots ,m)$$

whose connection weight with each neuron in the pattern layer is 1, and the other is

(10) $$S_{N}\;=\;\sum_{i=1}^{n}y_{i}p_{i}$$

whose connection weight is each factor *y_i_* of the output sample in the pattern layer in which the weighted sum is adopted to work out the summation of the output of corresponding neurons. The output of neurons in the output layer is

(11) $$Y\;=\;\frac{S_{N}}{S_{D}}$$

**Figure 2.**
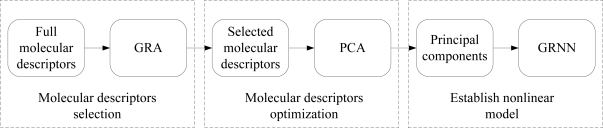
Flow chart of GP-GRNN model calculation (GRA, grey relational analysis; PCA, principal component analysis).

*i.e.*, the output of network. In our paper, Figure 2 shows a flow chart of GP-GRNN model calculation.

## 3. Computational

### 3.1. Data Set

Ninety-two (92) important organic carrier molecules containing NO are studied in this work. They are the four typical NO carrier molecules in the acetonitrile solution: *N*-nitrosamine compounds, *O*-nitrite, *C*-nitroso compounds, *S*-nitrosylation compounds (their molecular structures are shown in Table 1). The data set is randomly divided into a training set (80 molecules) and a test set (12 molecules). The training set used to adjust the model parameters and the test set is used to test the model's predictive ability.

### 3.2. Calculation of Molecular Descriptors

All of the calculations were done using Gaussian03 [40]. The geometry was optimized at the DFT/B3LYP level with 6-31G (d) basis set. Subsequently, vibrational frequencies were performed at the same theoretical level to confirm their local minima. The gas phase homolysis BDE is defined as the enthalpy change of the Equation 1 at 298 K in a vacuum [41]. The enthalpy of formation for each species was calculated using the following equation:

(12) $$H_{298}\;=\;E\;+\;ZPE\;+\;\Delta H_{298-0}\;+\;RT$$

The zero point energy correction was taken into account in the calculation. Δ*H*_298–0_ is the standard temperature correction term including *H*_vib_, *H*_rot_ and *H*_trans_.

(13) $$\Delta H_{\mathrm{hom}o}\;=\;H_{298(X•)}\;+\;H_{298(NO•)}\;-\;H_{298(X-NO)}$$

A set of molecular descriptors can be obtained from the geometry optimization and frequency calculations. The molecular descriptors include the calculated homolysis BDE value (Δ*H*_homo_), the net charge (*Q*_Y_) on the Y(C, N, O, S) atom which is bonded with the NO, the net charge (*Q*_N_) and (*Q*_O_) on the atoms N, O in the NO molecule fragment, the number of electrons (*N*_X_) on the fragment *X* (excluding the NO radical), the molecule dipole moment (μ) and the molecule polarizability (α), the highest occupied molecular orbital and the lowest unoccupied molecular orbital energy (*E*_HOMO_) and (*E*_LUMO_), the second highest occupied molecular orbital and the second lowest unoccupied molecular orbital energy (*E*_HOMO−1_) and (*E*_LUMO+1_), the energy gap (Δ*E*) between *E*_HOMO_ and *E*_LUMO_. The molecular descriptors can reflect covalent and ionic interactions in a molecule bond.

## 4. Results and Discussion

### 4.1. Calculation of Descriptor

For the 92 organic molecules containing the Y-NO (Y = C, N, O, S) bond, the B3LYP function is used to optimize the geometry at 6-31G (d) basis set level and the frequency is calculated to confirm the stable structure. Finally, the homolysis BDE of Y-NO (Y = C, N, O, S) bond and relevant molecular descriptors are obtained and shown in Table 2.

In addition, Table 2 also provides the homolysis BDE values that are observed in the experiment in the acetonitrile solution. The higher the homolysis BDE is, the stronger that the NO can bond with the carrier and *vice versa*. Lower homolysis BDE indicates that the carrier can serve as a good NO releasing agent. The four types of carrier molecules studied in this paper have lower homolysis BDE and are excellent NO radical carriers in the body. From Table 2, we can see that the carrier molecules containing N-NO, O-NO, S-NO and C-NO bonds have homolysis BDE at 12.4–43.8 kcal mol^−1^, 32.5–38.6 kcal mol^−1^, 17.2–29.2 kcal mol^−1^ and 27.5–31.4 kcal mol^−1^ respectively. The carrier molecule containing the S-NO bond has the lowest homolysis BDE which indicates that the carrier containing the S-NO bond is the best free NO radical carrier among the four compounds studied. The theoretical calculated homolysis BDE levels for the four compounds are at 12.63–35.57 kcal mol^−1^, 29.80–40.39 kcal mol^−1^, 17.49–27.96 kcal mol^−1^ and 17.69–24.62 kcal mol^−1^ respectively. Compared with the experimental values, the calculated value for the molecule carrier containing the N-NO bond is close to its experimental value with some underestimations and the calculated energy for the molecule carrier containing the O-NO and S-NO bonds is very close to their experimental values. The calculated energy for the molecule carrier containing the C-NO bond is lower than its experimental value.

### 4.2. Calculation Results of GRA

The experimenatal value of homolysis BDE of the 92 carrier molecules are used as the reference array. The 12 computed molecular descriptors are used as the contrast array. The closer the relation is to 1, the more relations the two arrays have and *vice versa*.

When the distinguishing coefficient is set to 0.5, Δ*H*_homo_, *Q*_Y_, *Q*_N_, *Q*_O_, *N*_X_, µ, α, *E*_HOMO−1_, *E*_HOMO_, *E*_LUMO_, *E*_LUMO+1_, Δ*E* are 0.8902, 0.6137, 0.7946, 0.8626, 0.8079, 0.7899, 0.8096, 0.8889, 0.8925, 0.8359, 0.7020, 0.8827 respectively. The relational coefficients of the 12 parameters on the experimental values in decreasing order are *E*_HOMO_, Δ*H*_homo_, *E*_HOMO−1_, Δ*E*, *Q*_O_, *E*_LUMO_, α, *N*_X_, *Q*_N_, μ, *E*_LUMO+1_, *Q*_Y_. Clearly, the *E*_HOMO_ and *E*_HOMO−1_ have a big impact on the homolysis BDE. The E_LUMO_ has a certain influence on the homolysis BDE. The E_LUMO+1_ has little impact on the homolysis BDE. The relational coefficient is 0.8902 which indicates a good match between the theoretical calculations and the experimental values. The difference between the E_HOMO_ energy level and E_LUMO_ energy level ΔE can measure the stability of the molecules and has a large impact on the homolysis BDE. In the NO molecular fragments, the oxygen’s electronegativity is greater than that of nitrogen and the net charge of oxygen has a larger impact than that of the nitrogen on the homolysis. The net charge on the Y(C, N, O, S) atoms which are connected to NO has the minimal impact on the homolysis which implies that the ionicity of chemical bonds is smaller. The polarization rate can measure the molecular deformation and affect the homolysis BDE. The number of electrons on the molecular fragment *X* which is connected to NO has a certain impact on the homolysis. The dipole moment is a vector that has little impact on the homolysis which indicates that the Y-NO direction has little impact on the molecular dipole moment.

### 4.3. Calculation Results of PCA

According to the results of GRA, the molecular descriptors that have a relation of greater than 0.8 are selected. The first eight molecular descriptors are used as the basic characteristics. A correlation matrix is generated after the correlation analysis on the eight molecular descriptors (Table 3). It can be seen that there is a certain correlation between them and the correlation between α and *N*_X_ is as high as 0.9331. It is inevitable that increasing the complexity of data analysis, if these eight selected molecular descriptors are taken as the final attribute characteristics directly, there would be some problems such as instable calculation and ill-conditioned matrix which are caused by superposed information existing in the above eight molecular descriptors. Nevertheless, these problems can be avoided through PCA that can also make the weight distribution of the molecular descriptors more reasonable, avoid the redundant information, and eliminate the not useful information.

The PCA is performed on the eight selected molecular descriptors to obtain the eigenvalues, variance and cumulative variance contribution rate for each principle component (Table 4). It can be seen that the first six principal components can explain 99.63% of the total variance of all variables, which means that the six indicators of the new indicators system can reflect 99.63% differences in samples. The eigenvalue for the seventh principal component is already very small and the eighth eigenvalue is 0. The smaller the eigenvalue is, the less amount of information its principal component contains. Therefore, the seventh principal component contains very little useful information and the eighth principal component contains no useful information. Therefore, the first six principal components are selected to avoid redundancy of information and eliminate the interference information, namely no useful information.

The weights of the first six principal components and the corresponding molecular descriptors are shown in Table 5. From the analysis of the weights of the first six principal components, it can be seen that the *E*_HOMO−1_ and *E*_LUMO_ have a higher load in the first principal component, the Δ*H*_homo_ and Δ*E* on the second principal component have a higher load, the *N*_X_ and α on the third principal component have a higher load, the Δ*H*_homo_ on the fourth principal component has a higher load, the *Q*_O_ on the fifth principal component has a higher load and the *E*_HOMO_ on the sixth principal component has a higher load. Thus the assignment to the weights has the theoretical basis. Meanwhile, the variance between either two of six principal components is 0 which means that the six principal components are unrelated to each other. Therefore, the instability and ill-conditioned matrix problems can be avoided in the computation.

After the GRA and PCA approaches are performed for the molecular descriptors selection and optimization, the first six principal components are used as the final GRNN inputs. To assess the GP-GRNN approach’s effect on calculating the homolysis BDE of 92 organic molecules, we compare the GP-GRNN correction results with the B3LYP/6-31G (d) correction results, the correction results of the full-descriptor GRNN without GRA and PCA (F-GRNN) and with GRA (G-GRNN), respectively. The differences between the experimental and calculated homolysis BDE for the F-GRNN, G-GRNN and GP-GRNN correction results are tabulated in Table 6.

**Figure 3.**
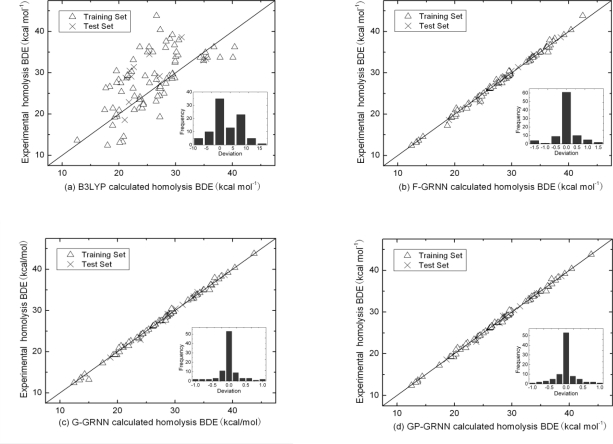
Calculated homolysis BDE *versus* experimental homolysis BDE for all 92 organic molecules; (**a**) B3LYP 6-31G (d) calculated homolysis BDE from the DFT approach; (**b**) full-descriptor GRNN corrected homolysis BDE for the F-GRNN approach; (**c**) The combined GRA and GRNN corrected homolysis BDE for the G-GRNN approach; (**d**) The combined GRA, PCA and GRNN corrected homolysis BDE for the GP-GRNN approach. Triangles (Δ) are for the training set and crosses (×) are for the test set. Insets are the histograms for the differences between the experimental and calculated homolysis BDE; All values are in units of kcal mol**^−^**^1^.

Figure 3a is the scatter diagram of the B3LYP/6-31G (d) results and experimental results. The vertical coordinates are the experimental values and the horizontal coordinates are the B3LYP/6-31G (d) calculated values. The diagonal line represents that the vertical coordinate and horizontal coordinates are equal. In Figure 3b, Figure 3c and Figure 3d the horizontal coordinates represent the correction results of F-GRNN, G-GRNN and GP-GRNN, respectively. It can be seen that the correction results of GP-GRNN are closer to the experimental values. The insets are the histogram for the deviation of three approaches in Figures 3a–3d. It is obvious that B3LYP/6-31G (d) approach has large systematic deviation while the F-GRNN, G-GRNN and GP-GRNN approaches corrected have small systematic deviation.

For GRNN, the initialization is to determine the study process of training samples. Then, the connection weight between the network structure and each neuron is determined after the determination of the study samples. The training process of network is just the process of determining *δ*. In the training process, the learning algorithm is to adjust the transfer function of each unit to acquire the best results of regression estimation by changing *δ*, not by adjusting the connection weight between neurons.

In the transfer function, the value of *δ* is increased progressively from 0.02 to 1 by the constant of the variation of 0.02. The optimal output of neural network can be decided in the process of the variation of *δ.* For F-GRNN, G-GRNN and GP-GRNN approaches, when the values of *δ* are respectively 0.18, 0.08 and 0.10, the best results of regression estimation appear. For the training test, the RMS before correction is 5.40 kcal mol^−1^, and the RMS of F-GRNN and G-GRNN after correction is 0.48 and 0.38 kcal mol^−1^; however the RMS of GP-GRNN is 0.30 kcal mol^−1^. For the test set, the RMS respectively decreases from 4.69 to 0.55, 0.46 and 0.39 kcal mol^−1^ (Table 7). The GP-GRNN approach improved DFT calculation results in both the training set and the test set separately. After the correction of GP-GRNN, the deviation between the value of each sample and the experimental value of homolysis BDE in the test set is reduced. Nevertheless, two deviations (in sample 22 and 24) are much bigger than the other ten. The reasons why the above two deviations are much bigger lie in the following two facts. (1) When the smoothing factor *δ* is larger, the network output Y (predictive value of homolysis BDE) approaches the mean of experimental value of homolysis BDE in all samples. On the contrary, when the smoothing factor *δ* tends to 0, Y is close to training sample. When the points which need predicating are included in the training sample, the predictive value of the experimental value of homolysis BDE calculated by using Equation 11 approaches corresponding experimental value of homolysis BDE in sample. Nevertheless, the predictive results may be worse if there are some sample points excluded in the sample. The value of *δ* is not the larger the better, nor the smaller the better. When the value of *δ* is moderate, all the experimental value of homolysis BDE in the training sample are taken into account and the experimenatal value of homolysis BDE corresponding to the sample points close to the predictive points add more weight. Hence, the value of *δ* is selected in this paper after many experiments with the aim to acquire the best network output. (2) Sample data is few in the training set and the features cannot be extracted in the training procedure of neural network. The prediction accuracy of GP-GRNN approach can be further improved as more and better experimental data are available. The consistency between the training and test set implied that the GP-GRNN results could indeed predict the homolysis BDE with higher accuracy than F-GRNN and G-GRNN.

The B3LYP/6-31G (d) calculations are carried out to evaluate the homolysis BDE of the 92 organic molecules, and their overall resulting RMS from the experimental data is 5.31 kcal mol^−1^. Upon the traditional F-GRNN correction approach, the RMS of the calculated homolysis BDE of the 92 organic molecules is reduced from 5.31 to 0.49 kcal mol^−1^ for the B3LYP/6-31G (d) calculation. With the G-GRNN and GP-GRNN correction, the RMS is reduced from 5.31 to 0.39 and 0.31 kcal mol^−1^, respectively (Table 7).

From Table 7, it can be seen that the correction result acquired from G-GRNN is better than that acquired from F-GRNN. If the molecular descriptors are added to G-GRNN, the good fitting ability and the poor generalization ability appear on the training set and the test set, respectively. This denotes that over fitting happens with the F-GRNN. Similarly, the correction result from GP-GRNN is also better than that from G-GRNN. This better result is facilitated by PCA which can optimize selected molecular descriptors. From the above discussion, it can be deduced that the descriptors selection and optimization play a key role to obtain a perfect model, although GRNN theoretically shows a stronger capability of anti-redundancy.

## 5. Conclusions

GP-GRNN approach was successfully used to improve the homolysis BDE calculation’s accuracy. GRA is used to select the appropriate molecular descriptors. PCA is used to optimize the selection of molecular descriptors. GRNN is used to establish non-linear model. The GP-GRNN approach reduced the calculated RMS of 92 organic molecules from 5.31 to 0.31 kcal mol^−1^. Compared with the F-GRNN and G-GRNN, GP-GRNN is more feasible and effective. Further, the GP-GRNN correction on top of the B3LYP/6-31G (d) results is a better approach to correct homolysis BDE and can be used as the approximation of experimental results, when the experimental results are limited to measurement, with very high accuracy. GP-GRNN approach extends the B3LYP/6-31G (d)’s feasibility and applicability. The more experimental data the training set has, the more accurate the GP-GRNN approach will be. GP-GRNN approach can be not only used to calculate the homolysis BDE, but also it can be applied to calculate the heterolysis BDE, absorption energy, ionization energy, formation heat and so on. In summary, the GP-GRNN approach is an effective and predictive tool that can be used in the study of physical and chemical properties at the molecular level.

## Figures and Tables

**Table 1.**
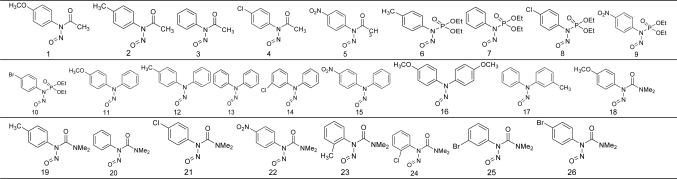

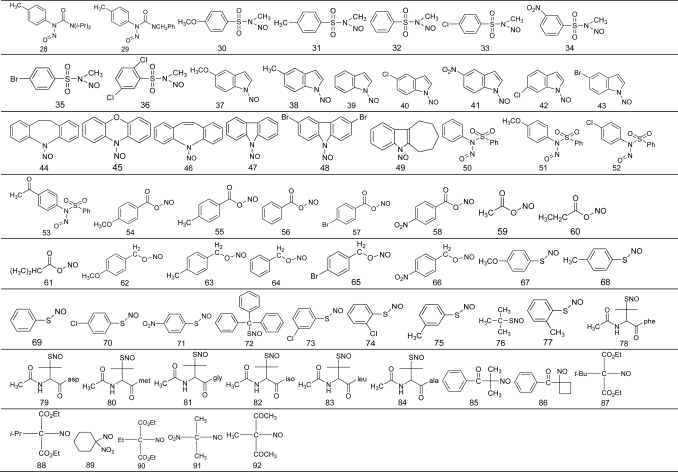
The structures of the 92 organic carrier molecules with Y-NO ^a^.

a 1–53 contain N-NO; 54–66 contain O-NO; 67–84 contain S-NO; 85–92 contain C-NO.

**Table 2.** Theoretical calculation of the Y-NO (Y = C, N, O, S) bond homolysis BDE (in the gas phase) and molecular descriptors ^a^.

**No. ^b^**	**Expt. ^c^**	**Δ*H*_homo_^d^**	***Q*_Y_**	***Q*_N_**	***Q*_O_**	***N*_X_**	**μ**	**α**	***E*_HOMO−1_**	***E*_HOMO_**	***E*_LUMO_**	***E*_LUMO+1_**	**Δ*E***
1	43.8	26.63	−0.309	0.222	−0.325	87	1.83	117.55	−0.2466	−0.2275	−0.0744	−0.0114	0.1532
2	36.1	28.22	−0.311	0.223	−0.324	79	0.91	111.89	−0.2493	−0.2457	−0.0761	−0.0190	0.1696
3	38.3	28.99	−0.312	0.224	−0.323	71	0.46	98.39	−0.2580	−0.2494	−0.0781	−0.0223	0.1713
4	37.6	28.31	−0.314	0.231	−0.324	87	1.53	111.82	−0.2584	−0.2542	−0.0855	−0.0344	0.1687
5	39.2	29.43	−0.318	0.236	−0.318	93	4.54	117.63	−0.2838	−0.2686	−0.1039	−0.0916	0.1648
6	34.5	25.37	−0.613	0.230	−0.341	145	2.81	182.34	−0.2432	−0.2360	−0.0556	−0.0086	0.1804
7	35	25.99	−0.614	0.231	−0.340	137	2.89	168.56	−0.2501	−0.2397	−0.0578	−0.0115	0.1819
8	36.2	23.67	−0.627	0.251	−0.336	153	3.65	182.09	−0.2476	−0.2432	−0.0666	−0.0196	0.1766
9	40.4	27.27	−0.615	0.239	−0.332	159	6.30	189.00	−0.2736	−0.2582	−0.0968	−0.0707	0.1614
10	36.2	25.30	−0.614	0.234	−0.338	171	3.60	190.40	−0.2498	−0.2425	−0.0652	−0.0231	0.1773
11	21.4	23.56	−0.247	0.213	−0.368	105	4.59	157.91	−0.2261	−0.2146	−0.0500	−0.0147	0.1646
12	21.4	24.10	−0.248	0.214	−0.366	97	3.78	152.40	−0.2278	−0.2213	−0.0523	−0.0184	0.1690
13	22.6	24.32	−0.250	0.216	−0.365	89	3.55	138.43	−0.2299	−0.2248	−0.0545	−0.0211	0.1702
14	24.1	23.71	−0.252	0.220	−0.361	105	2.73	150.61	−0.2373	−0.2318	−0.0628	−0.0309	0.1690
15	24.3	22.74	−0.256	0.226	−0.353	111	5.18	161.02	−0.2497	−0.2437	−0.0997	−0.0674	0.1440
16	21	22.69	−0.245	0.209	−0.376	121	5.80	178.22	−0.2209	−0.2028	−0.0445	−0.0135	0.1584
17	22.3	24.30	−0.249	0.215	−0.367	97	3.44	151.74	−0.2281	−0.2221	−0.0530	−0.0193	0.1691
18	28.3	19.93	−0.326	0.218	−0.338	103	3.29	136.91	−0.2379	−0.2228	−0.0567	−0.0045	0.1661
19	28.7	21.40	−0.327	0.219	−0.337	95	2.37	131.36	−0.2430	−0.2373	−0.0583	−0.0077	0.1791
20	29.1	22.17	−0.328	0.220	−0.336	87	2.54	117.73	−0.2507	−0.2402	−0.0604	−0.0108	0.1798
21	29.2	21.52	−0.330	0.226	−0.337	103	3.85	131.41	−0.2511	−0.2463	−0.0683	−0.0225	0.1781
22	33.1	22.52	−0.333	0.232	−0.331	109	6.56	137.77	−0.2664	−0.2592	−0.0970	−0.0752	0.1622
23	27.5	25.39	−0.322	0.219	−0.345	95	2.41	128.37	−0.2449	−0.2395	−0.0585	−0.0139	0.1810
24	23.1	26.55	−0.332	0.234	−0.334	103	1.55	127.84	−0.2527	−0.2444	−0.0631	−0.0253	0.1813
25	30.3	22.23	−0.330	0.228	−0.336	121	4.08	137.71	−0.2481	−0.2459	−0.0683	−0.0235	0.1776
26	29.4	21.50	−0.330	0.226	−0.337	121	3.75	139.32	−0.2492	−0.2441	−0.0682	−0.0230	0.1759
27	30.5	21.90	−0.330	0.227	−0.335	109	3.72	147.05	−0.2515	−0.2480	−0.0736	−0.0553	0.1744
28	26.6	18.38	−0.327	0.226	−0.348	127	3.31	174.50	−0.2394	−0.2322	−0.0553	−0.0030	0.1769
29	25.4	20.43	−0.322	0.218	−0.338	175	2.07	247.88	−0.2389	−0.2371	−0.0619	−0.0130	0.1752
30	33.7	35.57	−0.489	0.218	−0.367	105	5.11	128.42	−0.2500	−0.2435	−0.0595	−0.0395	0.1840
31	33.4	35.37	−0.492	0.221	−0.363	97	5.10	122.36	−0.2690	−0.2467	−0.0633	−0.0466	0.1833
32	34.9	35.23	−0.493	0.221	−0.361	89	4.54	108.58	−0.2783	−0.2491	−0.0662	−0.0499	0.1829
33	33	34.91	−0.495	0.220	−0.356	105	3.11	122.51	−0.2757	−0.2545	−0.0730	−0.0591	0.1815
34	33.9	34.64	−0.497	0.220	−0.349	111	2.00	125.62	−0.2986	−0.2629	−0.1125	−0.0797	0.1505
35	33	34.92	−0.495	0.221	−0.356	123	3.23	130.46	−0.2702	−0.2542	−0.0729	−0.0591	0.1813
36	33.7	34.32	−0.501	0.228	−0.349	121	3.73	132.97	−0.2716	−0.2568	−0.0733	−0.0694	0.1835
37	28.7	29.86	−0.219	0.233	−0.355	77	4.28	117.41	−0.2290	−0.2198	−0.0760	−0.0191	0.1438
38	28.6	29.36	−0.221	0.235	−0.351	69	3.13	111.26	−0.2372	−0.2276	−0.0779	−0.0198	0.1497
39	29	29.29	−0.223	0.237	−0.348	61	2.85	97.11	−0.2465	−0.2297	−0.0802	−0.0233	0.1495
40	29.8	29.44	−0.224	0.241	−0.343	77	2.20	111.05	−0.2467	−0.2414	−0.0893	−0.0360	0.1521
41	29.3	28.89	−0.229	0.249	−0.329	83	4.00	116.51	−0.2715	−0.2541	−0.1089	−0.0828	0.1451
42	28.47	28.43	−0.225	0.243	−0.341	77	3.79	110.11	−0.2569	−0.2338	−0.0891	−0.0356	0.1447
43	29.66	29.40	−0.224	0.241	−0.343	95	2.19	119.13	−0.2433	−0.2410	−0.0893	−0.0360	0.1518
44	22.9	21.76	−0.265	0.217	−0.369	103	3.79	155.37	−0.2333	−0.2254	−0.0486	−0.0139	0.1767
45	13.6	12.63	−0.230	0.208	−0.376	95	3.04	143.30	−0.2346	−0.2119	−0.0623	−0.0231	0.1496
46	19.2	19.23	−0.271	0.222	−0.369	101	3.70	165.31	−0.2299	−0.2158	−0.0573	−0.0476	0.1585
47	27.4	28.27	−0.210	0.229	−0.362	87	3.19	142.90	−0.2329	−0.2307	−0.0751	−0.0406	0.1557
48	28.3	26.63	−0.212	0.233	−0.353	155	0.72	189.38	−0.2452	−0.2386	−0.0898	−0.0596	0.1488
49	29.7	26.29	−0.209	0.223	−0.362	99	3.44	152.06	−0.2377	−0.2152	−0.0721	−0.0161	0.1431
50	13.2	20.67	−0.516	0.240	−0.332	121	5.38	156.76	−0.2605	−0.2475	−0.0697	−0.0523	0.1778
51	12.4	18.00	−0.513	0.237	−0.335	137	6.62	176.96	−0.2454	−0.2315	−0.0661	−0.0483	0.1654
52	13.1	20.13	−0.517	0.242	−0.331	137	4.85	171.04	−0.2594	−0.2532	−0.0759	−0.0586	0.1772
53	14.5	20.83	−0.518	0.241	−0.329	143	3.23	186.80	−0.2584	−0.2540	−0.0809	−0.0706	0.1732
54	32.5	29.88	−0.482	0.419	−0.205	79	4.05	111.15	−0.2672	−0.2370	−0.1077	−0.0420	0.1293
55	32.8	29.92	−0.483	0.421	−0.200	71	3.46	103.71	−0.2637	−0.2570	−0.1116	−0.0490	0.1454
56	33.9	30.02	−0.485	0.424	−0.195	63	2.84	89.12	−0.2682	−0.2658	−0.1150	−0.0522	0.1508
57	34.3	30.41	−0.489	0.427	−0.188	97	1.43	111.98	−0.2795	−0.2591	−0.1215	−0.0619	0.1375
58	38.6	31.03	−0.496	0.436	−0.171	85	2.96	107.57	−0.2964	−0.2920	−0.1352	−0.1074	0.1568
59	35	30.12	−0.491	0.421	−0.193	31	2.09	39.15	−0.3068	−0.2791	−0.1159	0.0010	0.1632
60	37.9	30.57	−0.488	0.420	−0.195	39	2.04	50.08	−0.3058	−0.2772	−0.1146	0.0031	0.1626
61	36.7	29.80	−0.488	0.420	−0.196	47	2.14	60.61	−0.3048	−0.2737	−0.1142	0.0011	0.1595
62	33.7	40.09	−0.380	0.383	−0.325	73	3.67	102.43	−0.2589	−0.2246	−0.0688	−0.0089	0.1559
63	33.7	37.82	−0.379	0.384	−0.323	65	3.06	96.97	−0.2564	−0.2425	−0.0706	−0.0160	0.1719
64	35	25.04	−0.380	0.385	−0.322	57	2.68	83.49	−0.2591	−0.2515	−0.0725	−0.0192	0.1790
65	36.2	40.39	−0.382	0.388	−0.318	91	1.54	104.40	−0.2738	−0.2482	−0.0785	−0.0326	0.1698
66	36.2	36.75	−0.388	0.394	−0.308	79	3.17	102.33	−0.2882	−0.2792	−0.0977	−0.0898	0.1815
67	21	17.49	0.289	0.054	−0.229	73	3.68	111.93	−0.2520	−0.2181	−0.0905	−0.0398	0.1276
68	21.4	18.94	0.297	0.054	−0.225	65	2.91	105.93	−0.2532	−0.2279	−0.0942	−0.0439	0.1337
69	19.4	19.67	0.300	0.055	−0.222	57	2.36	91.82	−0.2556	−0.2333	−0.0973	−0.0465	0.1360
70	19.2	19.25	0.296	0.062	−0.214	73	0.52	106.01	−0.2618	−0.2381	−0.1047	−0.0568	0.1333
71	18.6	21.03	0.303	0.075	−0.197	79	3.05	113.42	−0.2737	−0.2576	−0.1206	−0.1006	0.1370
72	23.4	23.60	0.318	0.012	−0.245	145	2.85	221.05	−0.2407	−0.2280	−0.0898	−0.0303	0.1382
73	20.9	20.02	0.298	0.065	−0.211	73	2.08	104.13	−0.2624	−0.2415	−0.1058	−0.0575	0.1357
74	19.3	27.21	0.292	0.077	−0.212	73	3.13	102.84	−0.2606	−0.2369	−0.1006	−0.0593	0.1363
75	19.9	19.54	0.302	0.053	−0.224	65	2.62	104.38	−0.2536	−0.2303	−0.0953	−0.0443	0.1351
76	25	27.96	0.316	−0.005	−0.247	49	2.76	73.43	−0.2584	−0.2331	^−^0.0887	^−^0.0126	0.1445
77	17.2	18.89	0.310	0.057	−0.231	73	3.50	108.44	−0.2391	−0.2188	^−^0.0870	^−^0.0415	0.1318
78	24.4	27.17	0.310	0.013	−0.243	179	2.14	218.16	−0.2537	−0.2397	−0.0944	−0.0275	0.1452
79	24.3	26.82	0.317	0.009	−0.236	161	4.93	172.55	−0.2702	−0.2519	−0.1066	−0.0373	0.1453
80	26.2	27.04	0.306	0.016	−0.240	171	2.49	197.57	−0.2432	−0.2241	−0.0977	−0.0288	0.1263
81	26.1	27.27	0.313	0.005	−0.242	131	2.22	148.15	−0.2537	−0.2427	−0.0974	−0.0254	0.1453
82	26.6	27.28	0.325	0.003	−0.239	163	4.86	191.09	−0.2583	−0.2448	−0.0994	−0.0299	0.1454
83	29.2	27.17	0.311	0.012	−0.243	163	1.57	190.12	−0.2535	−0.2395	−0.0942	0.0246	0.1452
84	27.4	27.16	0.306	0.017	−0.241	139	1.23	158.73	−0.2539	−0.2394	−0.0939	−0.0252	0.1455
85	28.8	21.17	−0.021	0.126	−0.284	79	2.14	112.96	−0.2611	−0.2274	−0.0916	−0.0653	0.1358
86	29.2	24.62	−0.036	0.139	−0.284	85	2.66	121.41	−0.2621	−0.2295	−0.0946	−0.0621	0.1349
87	27.5	20.34	−0.120	0.173	−0.254	117	3.29	135.55	−0.2727	−0.2310	−0.0915	−0.0139	0.1396
88	27.6	19.60	−0.119	0.151	−0.255	109	0.96	126.38	−0.2794	−0.2307	−0.0931	−0.0111	0.1376
89	26.2	22.5	0.187	0.136	−0.244	69	4.52	82.90	−0.2974	−0.2483	−0.1097	−0.0745	0.1386
90	30.4	19.55	−0.121	0.149	−0.262	101	2.94	115.49	−0.2788	−0.2314	−0.0927	−0.0150	0.1387
91	31.4	22.63	0.177	0.144	−0.239	47	3.99	55.09	−0.3002	−0.2542	−0.1127	−0.0783	0.1415
92	26.3	17.69	−0.118	0.139	−0.261	61	1.77	78.22	−0.2648	−0.2386	−0.1020	−0.0552	0.1366

a Unit: Δ*H*_homo_ (kcal mol^−1^), Charge (e), Dipole Moment (debye), Polar (a.u.) and Energy (a.u.);

b 1–53 contain N-NO, 54–66 contain O-NO, 67–84 contain S-NO, 85–92 contain C-NO;

c Measured in CH_3_CN at 25 °C by titration calorimentry;

d The calculated homolysis BDE are with zero-point energy (ZPE) and thermal corrections to enthaply at 298 K by B3LYP/6-31G (d).

**Table 3.** Correlation matrix between the selected molecular descriptors.

	**Δ*H*_homo_**	***Q*_O_**	***N*_X_**	**α**	***E*_HOMO−1_**	***E*_HOMO_**	***E*_LUMO_**	**Δ*E***
Δ*H*_homo_	1.0000	−0.0712	−0.1201	−0.2483	−0.3506	−0.4384	−0.1329	0.2507
*Q*_O_		1.0000	−0.2639	−0.3599	−0.5379	−0.3361	−0.8101	−0.6399
*N*_X_			1.0000	0.9331	0.3038	0.1239	0.2756	0.2088
α				1.0000	0.5355	0.3294	0.4073	0.1729
*E*_HOMO−1_					1.0000	0.7963	0.7207	0.1078
*E*_HOMO_						1.0000	0.5695	−0.2589
*E*_LUMO_							1.0000	0.6465
Δ*E*								1.0000

**Table 4.** Eigenvalues and cumulative contributions of variances.

**No.**	**Eigenvalues**	**Variances (%)**	**Cumulative (%)**
1	3.7039	0.4630	0.4630
2	1.8642	0.2330	0.6960
3	1.3980	0.1747	0.8708
4	0.6259	0.0782	0.9490
5	0.2211	0.0276	0.9766
6	0.1573	0.0197	0.9963
7	0.0296	0.0037	1.0000
8	0.0000	0.0000	1.0000

**Table 5.** First six principal components of the weight coefficients and the corresponding molecular descriptors.

**No.**	**Δ*H*_homo_**	***Q*_O_**	***N*_X_**	**α**	***E*_HOMO−1_**	***E*_HOMO_**	***E*_LUMO_**	**Δ*E***
1	−0.1532	−0.3985	0.3084	0.3863	0.4423	0.3413	0.4597	0.2235
2	−0.5035	−0.3290	0.0001	−0.0929	−0.2155	−0.4219	0.1850	0.6090
3	0.0213	−0.2076	−0.6685	−0.5414	0.1997	0.3165	0.2750	0.0294
4	0.8325	0.0471	−0.0684	−0.0798	−0.1917	−0.3043	0.0950	0.3940
5	−0.1589	0.7964	−0.0431	0.0081	0.4025	−0.0457	0.2491	0.3352
6	0.0479	−0.2266	−0.2212	0.1074	0.6798	−0.4933	−0.4229	−0.0391

**Table 6.** The experimental homolysis BDE values and the differences between the experimental and calculated values of 92 organic molecules (in kcal mol^−1^).

**No.**	**Expt. ^a^**	**Deviation ^b^**	**Deviation ^c^**	**Deviation ^d^**	**Deviation ^e^**
1	43.80	17.17	1.35	−0.08	0.01
2	36.10	7.88	−0.47	0.28	−0.30
3	38.30	9.31	0.76	−0.29	0.29
4	37.60	9.29	0.26	0.01	0.02
5	39.20	9.77	0.04	0.00	0.01
6^f^	34.50	9.13	0.66	−0.61	0.10
7	35.00	9.01	−0.11	0.01	−0.01
8	36.20	12.53	0.15	−0.03	0.01
9	40.40	13.13	0.01	0.00	0.00
10	36.20	10.90	0.05	0.00	0.01
11	21.40	−2.16	−0.22	0.08	−0.06
12	21.40	−2.70	−0.73	0.53	−0.56
13	22.60	−1.72	0.18	−0.29	0.33
14	24.10	0.39	0.07	−0.05	0.06
15	24.30	1.56	−0.01	0.00	0.00
16	21.00	−1.69	−0.02	0.00	−0.01
17	22.30	−2.00	0.04	−0.33	0.30
18	28.30	8.37	1.01	−0.05	0.01
19	28.70	7.30	0.58	−0.11	0.05
20^f^	29.10	6.93	0.29	−0.03	0.67
21	29.20	7.68	−0.24	−0.13	−0.20
22	33.10	10.58	0.01	0.00	−0.01
23	27.50	2.11	0.18	−0.29	0.50
24	23.10	−3.45	−1.43	0.61	−0.59
25	30.30	8.07	0.65	−0.87	0.49
26	29.40	7.90	−0.16	−0.22	−0.10
27^f^	30.50	8.60	0.56	−0.42	0.13
28	26.60	8.22	−0.15	−0.02	0.07
29	25.40	4.97	−0.01	0.00	−0.01
30	33.70	−1.87	−0.01	0.00	0.00
31^f^	33.40	−1.97	0.05	−0.16	0.11
32	34.90	−0.33	0.08	0.00	0.00
33^f^	33.00	−1.91	−0.05	0.16	−0.11
34	33.90	−0.74	0.00	0.00	0.00
35	33.00	−1.92	−0.27	0.27	−0.25
36	33.70	−0.62	0.23	−0.27	0.25
37	28.70	−1.16	−0.03	0.00	0.01
38	28.60	−0.76	−0.42	0.10	−0.02
39	29.00	−0.29	−0.18	−0.03	0.02
40	29.80	0.36	0.05	−0.05	0.06
41	29.30	0.41	−0.03	0.00	0.00
42	28.47	0.04	−0.13	0.04	−0.01
43	29.66	0.26	−0.08	0.02	0.03
44^f^	22.90	1.14	−1.45	1.09	−0.87
45	13.60	0.97	−0.06	0.00	0.00
46	19.20	−0.03	−0.10	0.03	−0.01
47	27.40	−0.87	−0.12	0.01	−0.11
48	28.30	1.67	0.00	0.00	0.00
49	29.70	3.41	0.08	0.00	−0.01
50	13.20	−7.47	−0.07	1.82	−0.38
51	12.40	−5.60	−0.01	0.02	−0.10
52	13.10	−7.03	−0.11	0.26	−0.12
53	14.50	−6.33	0.09	−0.23	0.12
54	32.50	2.62	−0.01	−0.26	0.10
55	32.80	2.88	−0.24	0.04	−0.05
56	33.90	3.88	0.25	−0.04	0.05
57	34.30	3.89	0.01	0.00	0.01
58^f^	38.60	7.57	0.00	0.00	0.01
59	35.00	4.88	−1.38	1.01	−0.97
60	37.90	7.33	1.27	−1.03	1.00
61	36.70	6.90	−0.08	0.24	−0.25
62	33.70	−6.39	0.01	0.00	0.00
63	33.70	−4.12	−0.01	0.00	0.00
64	35.00	9.96	0.01	0.02	−0.01
65	36.20	−4.19	0.01	0.00	0.00
66	36.20	−0.55	−0.01	0.00	0.00
67	21.00	3.51	0.89	−0.17	−0.01
68	21.40	2.46	1.12	−0.81	0.87
69	19.40	−0.27	−0.65	0.38	−0.42
70	19.20	−0.05	−0.24	0.66	−0.65
71^f^	18.60	−2.43	−0.36	0.13	−0.01
72	23.40	−0.20	−0.13	0.00	−0.01
73	20.90	0.88	0.46	−0.66	0.64
74	19.30	−7.91	−0.13	0.26	−0.10
75	19.90	0.36	−0.31	0.44	−0.37
76	25.00	−2.96	0.01	0.00	0.01
77	17.20	−1.69	−1.50	0.21	−0.16
78	24.40	−2.77	−0.01	0.05	−0.12
79	24.30	−2.52	−0.46	0.04	−0.05
80	26.20	−0.84	0.12	0.00	0.01
81	26.10	−1.17	0.01	0.00	0.00
82	26.60	−0.68	0.47	−0.09	0.17
83^f^	29.20	2.03	0.33	−0.49	0.44
84^f^	27.40	0.24	−0.33	0.49	−0.45
85^f^	28.80	7.63	0.08	−0.04	−0.01
86	29.20	4.58	0.01	0.00	0.00
87	27.50	7.16	−0.80	0.17	−0.07
88	27.60	8.00	−0.13	0.77	−0.76
89	26.20	3.70	0.01	0.00	0.00
90	30.40	10.85	0.87	−1.02	0.86
91^f^	31.40	8.77	0.29	−0.09	0.01
92	26.30	8.61	−0.01	−0.01	0.01

a Experimental data;

b Differences between the B3LYP calculated and experimental values;

c Differences between calculated and experimental values for DFT-F-GRNN calculation;

d Differences between calculated and experimental values for DFT-G-GRNN calculation;

e Differences between calculated and experimental values for DFT-GP-GRNN calculation;

f Organic molecules belong to the test set.

**Table 7.** RMS of B3LYP/6-31G (d), DFT-F-GRNN, DFT-G-GRNN, and DFT-GP-GRNN correction (in kcal mol^−1^).

	**B3LYP/6-31G (d)**	**F-GRNN**	**G-GRNN**	**GP-GRNN**
Training set	5.40	0.48	0.38	0.30
Test set	4.69	0.55	0.46	0.39
Overall	5.31	0.49	0.39	0.31
